# Acute Psychotic Episode Induced by Antimicrobial Treatment

**DOI:** 10.1155/2023/9996763

**Published:** 2023-04-01

**Authors:** Savera I. Arain, Majed Al Shakhori, Shabeer A. Thorakkattil, Omer Amin

**Affiliations:** ^1^Pharmacy Services Department, Johns Hopkins Aramco Healthcare (JHAH), Saudi Arabia; ^2^Psychiatry Department, Johns Hopkins Aramco Healthcare (JHAH), Saudi Arabia

## Abstract

Psychosis is an abnormal state of mind that leads to losing touch with reality. Symptoms may include delusions and hallucinations, amongst other features. Psychosis is known to increase the risk of other health conditions and may have serious adverse outcomes. This is a case report of a 26-year-old woman with no previous psychiatric history who presented with symptoms and signs suggestive of acute psychosis shortly after starting a postprocedural combination of antimicrobials. The patient's family decided to stop the antimicrobials as they observed an escalation of the psychotic symptoms with the ongoing use of antibiotics. The patient was subsequently brought to the emergency service (EMS) department, and she was admitted to the behavioral health unit. The treatment team managed to stabilize the patient with several interventions, including the administration of antianxiety and antipsychotic medications along with psychosocial intervention. The symptoms of psychosis resolved within 3-4 days, and she was discharged home. Even though transient psychotic episodes have been reported previously with antibiotics, this case emphasizes the increased need for vigilance and reporting in patients receiving antimicrobials.

## 1. Introduction

Psychosis is a mental health condition characterized by loss of contact with reality. During a psychotic episode, the patients experience unusual, unpleasant, and sometimes frightening thoughts and feelings. They lose the ability to differentiate between real vs. their own imagination. Also, psychosis can have an acute onset, or it can start gradually. The symptoms include delusions, hallucinations in various modalities, inappropriate behavior, incoherent speech, thought disorder, sleep problems, social withdrawal, lack of motivation, and difficulties carrying out activities of daily living. While hallucinations are where someone sees, hears, smells, tastes, or feels things that do not exist outside their mind, delusion is an unshakably false belief indicating abnormal thoughts [1, 2]. Psychosis can be caused by psychological conditions such as Schizophrenia, bipolar disorder, and severe depression, which are considered serious and enduring mental illnesses. However, psychosis can also be caused by stressful life events, misuse of alcohol and drugs, some neurological conditions, childbirth, disrupted sleep patterns, or certain prescription medications [3]. Early interventions to treat psychosis are essential as these patients may harm themselves. Also, while the outcomes vary, most patients have shown a better and more effective response to treatment when treated early with an appropriate combination of medication and psychosocial support therapy [4, 5].

Acute psychosis and other adverse neuropsychiatric adverse events from antibiotics have previously been reported and well documented, with evidence of the direct relationship between acute psychosis and antimicrobial use [6, 7]. Studies show that patients with a mental disorder are more likely to receive more than one antibiotic during their hospital stay, and while antimicrobials have been linked to neuropsychiatric adverse events, the underlying mechanism for this association is still unknown [8, 9]. Presented here is a case report of a 26-year-old female patient who developed acute psychosis after the use of metronidazole and amoxicillin/clavulanate. Metronidazole and amoxicillin/clavulanate are vastly used antibiotics, and therefore it is important to continue reporting these “real” medical case reports as it will help improve early recognition and patient care.

It is therefore important to continue reporting these “real” medical case reports as it will help improve early recognition and patient care.

“We present the following case in accordance with the CARE guideline.”

## 2. Case Presentation

The patient is a 26-year-old female with no other significant medical history. She is married with 4 healthy children, and her family reports no current psychosocial stressors. She was brought to the EMS because of severe agitation, visual hallucinations, persecutory delusions, disturbed thinking, complete lack of sleep, and extreme mood changes. She was administered 5 mg of haloperidol, 25 mg of diphenhydramine, and 2 mg of lorazepam in the EMS, followed by admission to the hospital for further workup and observation. The patient's admission plan included a complete medical evaluation, including vitals, labs, computed tomography (CT) brain, and a consult for psychiatry.

The patients' medical workup did not reveal any abnormal findings. The patients' social, drug-use, medical, or personal history did not reveal any significant findings that could be linked to this acute psychotic episode. The patient does not have a family history of any mental illness.

The patient started to experience acute psychotic episodes 11 days before this admission to EMS. At that time, the patient was pregnant (end of the first trimester), and she started to experience vaginal bleeding while she was traveling by road to attend a family event in a distant city. She was accompanied by her spouse, who took her urgently to the nearest hospital, where she underwent dilation and curettage (D&C) due to miscarriage and was started on 2 antibiotics (amoxicillin-clavulanate and metronidazole). D&C was carried out without complications and did not require any postprocedure intervention such as blood transfusion. She was then discharged with advice to continue on both antibiotics for a total duration of 7 days. The patient started showing signs of paranoia, anxiety, excessive fear, olfactory, visual and auditory hallucinations, religious utterances, nightmares, and a complete lack of sleep, approximately 24-48 hours after starting antibiotic treatment. Based on her family's report, she was apprehensive about her well-being and repeatedly asked if she was going to die. She did not sleep or eat for 3 days. She resisted seeking medical care as she felt convinced that God will cure her. She stopped taking her antibiotics on day number 6 from the start date. As her condition worsened over the next day, she was brought to EMS and later transferred to the inpatient unit. Full medical workup including physical exam, electrocardiogram, urine drug test, labs, blood and urine cultures, and CT scan was completed and there were no significant findings. A complete absence of neurological pathology was noted, and there were no clinically relevant symptoms to necessitate the use of magnetic resonance imaging (MRI). A consultation with psychiatry was made and the patient was kept under close observation for behavioral changes. She was started on olanzapine 5 mg orally twice daily, olanzapine 5 mg orally every 8 hours as needed for agitation, and lorazepam 1 mg every 6 hours as needed for anxiety or agitation. She stayed at the hospital for 4 days, progressed to improve, and continued receiving psychosocial support. She slept well and remained cooperative with the treatment team. She was discharged home on olanzapine 10 mg at bedtime with a follow-up within a week. The family was advised to monitor the patient's mental health and return to EMS if the patient's condition worsened.

## 3. Discussion

This is a unique descriptive case report of an acute psychotic episode induced by short-term antimicrobial therapy post-D&C procedure. The risk of postprocedural infections from D&C can be lowered by using proper aseptic conditions; however, the procedure still carries the risk of infection. Thiyagarajan et al. compared the incident rate of infection in patient's post-D&C procedure. They found no statistical significance in infection rate post-D&C in the group of patients who received antibiotics vs. those who did not. Their study also highlighted the disadvantage of increasing antimicrobial resistance and the possibility of adverse drug reactions as a matter of concern for patients receiving prophylactic antibiotics for a minor procedure like D&C [10]. Metronidazole is one of the mainstay drugs for treating bacterial and parasitic infections and is often used in combination with other antibiotics as surgical prophylaxis [11, 12]. Clavulanic acid acts as a potent beta-lactamase inhibitor. Its combination with amoxicillin has proven extremely effective in treating upper and lower respiratory tract infections, urinary tract infections, skin and soft tissue infections, and obstetric, gynecological, and intra-abdominal infections [13].

Psychotic symptoms that are transient in nature as an adverse effect of antimicrobial treatment have been gaining attention for the past several years. There is evidence suggesting a direct relationship between acute psychosis and antibiotic exposure. “Hoigné syndrome” was named after the discovering clinician who noted psychotic reactions such as visual and auditory hallucinations and multiple delusions in patients being prescribed penicillin. While the exact mechanism for this correlation is still unknown and it varies between different antibiotic classes, there is the hypothesis that it could be due to the direct impact of antibiotics on neurotransmitter function or modulation of cytokine production. Cytotoxic and vasogenic edema and its inhibitory effect on the GABA receptor are all possible mechanisms associated with neurotoxicity symptoms in patients on metronidazole. Beta-lactam classes, including penicillin, cephalosporin, and carbapenems, have been reported to cause neurotoxicity by acting as GABA-A antagonist. The chemical structure of the beta-lactam ring is similar to the GABA antagonist bicuculline, which may lead to excitatory effects such as psychosis. The neuropsychiatric adverse effect of beta-lactam includes seizures, hallucinations, delirium, and psychosis [6, 7]. The psychotic or manic symptomology is also referred to as “antibiomania” and is defined as a condition where the patient develops psychotic symptoms, specifically mania, upon initiation of antibiotics and later returns to a normal state upon treatment discontinuation [6, 11, 14].

This patient did not report any other psychosocial stressor or another confounding factor at the onset of this acute psychotic episode. Differential diagnosis included ruling out metabolic issues such as hypoglycemia, electrolyte imbalance, system organ failure, physical trauma (head injury), dehydration, malnutrition, anemia, and delirium on the basis of clinical presentation, and laboratory results. The association of the acute psychotic episode was further supported by the complete resolution of symptoms upon antibiotic discontinuation. Also, it is important to note that there is no evidence of the definitive link between D&C and psychosis. Case reports of both metronidazole and amoxicillin/clavulanate have previously been reported for association with the onset of psychotic symptoms. This case is similar to previously reported cases as both the onset and resolution of psychotic symptoms occurred within 7 days of beginning and discontinuation of antimicrobial therapy, respectively. It can therefore be implied that antibiotic treatment has a strong correlation with the patient's onset of an acute psychotic episode. There is, however, a limitation to this report that the psychotic episode cannot be exclusively attributed to metronidazole or augmentin, as both medications were started and stopped simultaneously. Secondly, a rechallenge with either antibiotic was not done due to ethical considerations and risk vs. benefit calculation. More research is needed to find definitive underlying mechanisms for antibiotic-induced psychosis and to compare the number of reported cases for individual antimicrobials to help prescribers make a more informed decision for patients with previous or current psychiatric illnesses.

## 4. Conclusion

The antibiotics, especially when used in combination, need more careful prescribing and monitoring. Early and timely intervention to treat any psychiatric adverse effects from antimicrobials can make a significant impact on patients' overall health. The exact cause of psychiatric adverse effects from the use of antibiotics is still largely unknown. There is also a lack of evidence-based treatment guidelines for resolving neuropsychiatric side effects developed as a result of antimicrobial use. However, given that the type of medical treatment and recovery can be greatly influenced by early recognition of the psychiatric side effects, it is important to continue reporting all occurrences of antibiomania cases.

## Data Availability

The data will be available by contacting the corresponding using the provided email.

## References

[1] Kiran C., Chaudhury S. (2009). Understanding delusions. *Industrial Psychiatry Journal*.

[2] https://www.nhs.uk/mental-health/conditions/psychosis/symptoms/.

[3] https://www2.hse.ie/conditions/psychosis/causes/.

[4] https://www.nhs.uk/mental-health/conditions/psychosis/treatment/.

[5] Guvenek-Cokol P. E. *Psychosis: will catching early warning signs help?*.

[6] Essali N., Miller B. J. (2020). Psychosis as an adverse effect of antibiotics. *Brain, Behavior, & Immunity-Health*.

[7] (2019). *Psychiatric adverse effects of antibiotics*.

[8] Kehoe K. P., Miller B. J. (2020). Acutely ill psychiatric inpatients and antimicrobial *exposure*. *Psychiatry*.

[9] A link between antibiotics and mental illness?. https://www.hopkinsmedicine.org/news/publications/hopkins_medicine_magazine/medical_rounds/fall-2016/a-link-between-antibiotics-and-mental-illness.

[10] Thiyagarajan K., Chaudhary V., Patil Y., Gawali V. (2021). Is there any role of antibiotic as a post procedure prophylaxis in dilatation and curettage? A comparative single centre study at tertiary care centre. *International Journal of Reproduction, Contraception, Obstetrics and Gynecology*.

[11] Ly D., DeLisi L. E. (2017). Can antibiotics cause a psychosis?: case report and review of the literature. *Schizophrenia Research*.

[12] Weir C. B., Le J. K. (2022). Metronidazole. *StatPearls*.

[13] Ball P. (2007). The clinical development and launch of amoxicillin/clavulanate for the treatment of a range of community-acquired infections. *International Journal of Antimicrobial Agents*.

[14] https://wikem.org/wiki/Antibiomania.

